# The potential application of artificial intelligence in veterinary clinical practice and biomedical research

**DOI:** 10.3389/fvets.2024.1347550

**Published:** 2024-01-31

**Authors:** Olalekan Chris Akinsulie, Ibrahim Idris, Victor Ayodele Aliyu, Sammuel Shahzad, Olamilekan Gabriel Banwo, Seto Charles Ogunleye, Mercy Olorunshola, Deborah O. Okedoyin, Charles Ugwu, Ifeoluwa Peace Oladapo, Joy Olaoluwa Gbadegoye, Qudus Afolabi Akande, Pius Babawale, Sahar Rostami, Kehinde Olugboyega Soetan

**Affiliations:** ^1^Faculty of Veterinary Medicine, University of Ibadan, Ibadan, Nigeria; ^2^College of Veterinary Medicine, Washington State University, Pullman, WA, United States; ^3^Faculty of Veterinary Medicine, Usman Danfodiyo University, Sokoto, Nigeria; ^4^Department of Population Medicine and Pathobiology, College of Veterinary Medicine, Mississippi State University, Starkville, MS, United States; ^5^Department of Pharmaceutical Microbiology, University of Ibadan, Ibadan, Nigeria; ^6^Department of Animal Sciences, North Carolina Agricultural and Technical State University, Greensboro, NC, United States; ^7^Department of Physiology, University of Tennessee Health Science Center, Memphis, TN, United States; ^8^Department of Biological Sciences, University of Notre Dame, Notre Dame, IN, United States; ^9^Department of Pathobiological Sciences, School of Veterinary Medicine, Louisiana State University, Baton Rouge, LA, United States

**Keywords:** artificial intelligence (AI), veterinary clinical practice, biomedical research, science, machine learning

## Abstract

Artificial intelligence (AI) is a fast-paced technological advancement in terms of its application to various fields of science and technology. In particular, AI has the potential to play various roles in veterinary clinical practice, enhancing the way veterinary care is delivered, improving outcomes for animals and ultimately humans. Also, in recent years, the emergence of AI has led to a new direction in biomedical research, especially in translational research with great potential, promising to revolutionize science. AI is applicable in antimicrobial resistance (AMR) research, cancer research, drug design and vaccine development, epidemiology, disease surveillance, and genomics. Here, we highlighted and discussed the potential impact of various aspects of AI in veterinary clinical practice and biomedical research, proposing this technology as a key tool for addressing pressing global health challenges across various domains.

## 1 Introduction

Artificial intelligence (AI) refers to the ability of computerized systems or computer-controlled robots to execute tasks typically associated with intelligent beings. In essence, AI represents machine intelligence, emulating cognitive functions such as learning and problem-solving observed in humans and animals (1). AI emerged as an academic field in 1956 and has investigated numerous approaches, encompassing brain simulation, modeling human problem-solving, logic, knowledge databases, and the emulation of human behavior (2). Despite its recognition as an academic subject in 1956, AI remained a relatively insignificant scientific approach with limited practical applications for many years until recently. Veterinary medicine, a diverse field covering areas like pet health, public and population health, zoonotic diseases, and animal production, parallels AI's impact across various scientific domains such as philosophy, mathematics, neuroscience, control theory, cybernetics, computer engineering, and data sciences. The convergence of these two (2) expansive and evolving fields holds the promise of mutually influencing each other. The remarkable progress and possibilities in the development, application, and clinical integration of AI in human and animal healthcare present exciting prospects. In human healthcare, AI has been used effectively in precision medicine by tailoring treatment based on genomic variations and other factors, in drug development and discovery where DeepTox, a deep learning- based model assesses the toxic effects of compounds comprising drug molecules, in image-based diagnosis and image-guided surgery analysis by utilizing computer vision (3). Similarly, the advent of AI in Veterinary Medicine opens new avenues to enhance the wellbeing of animals and their caregivers. However, these promising opportunities also bring distinct challenges, particularly in comprehending, interpreting, and embracing such potent and emerging technology, given the rapid pace and dynamism of research and commercial product developments.

## 2 AI roles in veterinary clinical practice

**Figure 1:**
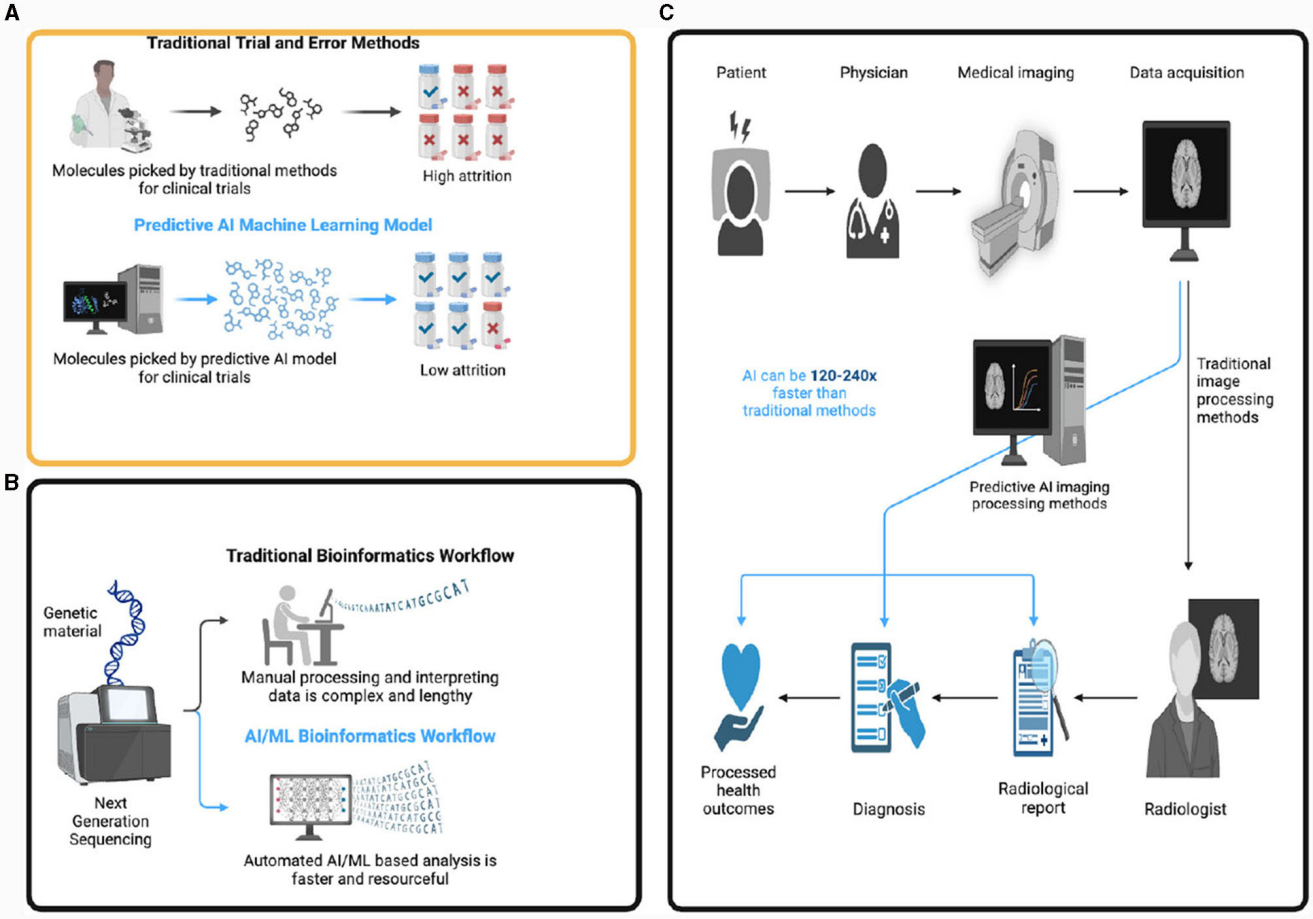
Schematic representation of the plausible use of artificial intelligence (AI) and machine learning (ML) in veterinary science. AI and ML are useful tools in the field of animal health surveillance, especially in the development of models that make predictions, and identify useful drug targets for development **(A)**. ML can also be used in the exploration of genome sequencing data **(B)**. Employing ML in whole genome sequencing improves source attribution, assessment of pathogenicity, prediction of antibiotic resistance phenotypes, and prediction of clinical outcomes of veterinary patients. Also, Radiomics, a cutting-edge aspect of precision medicine, utilizes advanced mathematical analyses to quantitatively approach medical imaging. **(C)** Radiomics encompasses the extraction of numerous features from images, converting them into quantitative data. These data, when subjected to sophisticated statistical tools, become integral to the progression of personalized precision medicine (PPM). The figure was created with BioRender.com.

AI holds the capacity to fulfill diverse functions in veterinary clinical practice, transforming the delivery of veterinary care and elevating outcomes for animals. As shown in Figure 1, Radiomics, a cutting-edge aspect of precision medicine, utilizes advanced mathematical analyses to quantitatively approach medical imaging. It suggests that medical images encapsulate information about disease-specific processes beyond human visual analysis. Radiomics encompasses the extraction of numerous features from images, converting them into quantitative data. These data, when subjected to sophisticated statistical tools, become integral to the progression of personalized precision medicine (PPM). Radiomics applications extend across various medical imaging modalities, including MRI, CT, PET, and ultrasound. Machine Learning (ML), a fundamental subfield of AI, assumes a crucial impact by crafting algorithms that learn from existing data to make accurate predictions for new data (4). The application of AI in animal health (AH) facilitates the management of highly complex subjects like quantitative and predictive epidemiology, precision-based therapeutics for animals and humans, and host-pathogen interactions. AI can contribute to disease diagnoses, precise diagnosis with minimal errors, comprehension of intricate biological interactions, proposal of solutions, and enhancement of risk assessment and target-specific interventions (5).

### 2.1 Disease diagnosis

Early disease detection is essential for effective therapy, allowing treatments that may enhance patient outcomes and prognosis. AI is essential in this context because it uses machine learning and complex algorithms to examine large medical datasets, such as imaging and clinical data. The application of AI techniques to veterinary medicine is crucial for addressing complex problems in the fields of numerical and predictive epidemiology, precision medicine for people and animals, and host-pathogen interaction. Continuous monitoring using AI-powered systems guarantees continued surveillance for at-risk patients, making it easier to quickly detect changes in health markers. This is particularly helpful for chronic illnesses since it enables early intervention and proactive management for the patients. According to a study by Reagen, a machine-learning algorithm has been used to diagnose chronic hypoadrenocortism (CHA) in dogs. Diagnosing CHA presents a considerable challenge due to limited awareness and a low index of suspicion. In Raegen's study, machine learning techniques were employed to assist in CHA diagnosis, utilizing routinely collected screening diagnostics such as complete blood count and serum chemistry panel (6). Texture Analysis (TA) has similarly been utilized to distinguish non-infectious inflammatory meningoencephalitis from canine glial cell neoplasia. This task can be very difficult to do largely as a result of overlapping image characteristics, even for seasoned diagnostic imaging specialists, as highlighted in the study by Wanamaker et al. (7). There are current efforts to integrate AI and radionics as tools to support decision-making and incorporate them into standard clinical processes and diagnostics, to enhance and improve sensitivity, accuracy, and reproducibility (3). A quantitative approach to medical imaging, known as “Radiomics” enhances data interpretation through the application of advanced and sometimes paradoxical mathematical analyses.

### 2.2 Zoonotic disease monitoring

AI tool is a promising tool in zoonotic disease monitoring and surveillance. Particularly, AI's potential rise in the medical field and veterinary research holds significant promise in addressing the problems associated with emerging zoonotic illnesses (8). Utilizing machine learning and sophisticated algorithm models, the integration of AI tools with traditional disease control tactics presents new opportunities for comprehending, forecasting and mitigating the effects of zoonotic illnesses (9). Many AI prediction models are available that can determine risk variables and project a person's probability of acquiring a disease, allowing for early detection and intervention (10). There are webservers and available AI models that have been used to predict host range susceptibility and viral host prediction. AI models such as Word2vec, a Natural language processing (NLP) model used to predict host ranges of Influenza virus by features vectorized from viral nucleotide and protein sequences (11). VIDHOP, a deep learning method for predicting viral hosts based on viral nucleotide sequences only (12). Webservers like FluSPred (Flu Spread Prediction), a machine learning based tool to predict the human associated Influenza A virus strains with the help of its protein and genome sequences, stating whether a viral strain has the potential to infect human hosts. It was developed the help prioritize high risk viral strains for future research, aid study the emergence or the risk a novel influenza virus possesses if it acquires the capability of human-to-human spread (13).

### 2.3 Epidemiology and surveillance

As per surveillance, AI can be leveraged in determining the ability to detect pathogens, helping research scientists and veterinarians prioritize samples or cases with the potential of being positive, thereby managing resources and laboratory capacities and maintaining focus on relevant samples. These methodologies have been consistently applied in animal disease surveillance, and food-borne diseases (14) making the most of the metadata associated with the biological samples or cases. Machine learning algorithms linked to online/web-based systems like PADI-web, and biosurveillance systems are examples of platforms that have been developed in recent times to detect and identify emerging animal diseases early. Also, ML algorithms have been used to detect the potential of utilizing genomic and epidemiological metadata simultaneously for food and water-borne disease surveillance (15).

### 2.4 Development of disease models

Machine learning (ML) is useful in the field of animal health surveillance, especially in the development of models that make predictions. For instance, farms that are more prone to becoming infected with a certain pathogen can be identified. This is garnered from previous case data and a set of potential risk factors. In Canada, ML models have been used in porcine epidemic diarrhea virus cases to predict future trends (16). Machine learning can be applied to decipher methods of the susceptibility of hosts to disease. For example, Becker et al. use ML to identify which bat species are potential reservoir hosts of betacoronaviruses. Wardeh et al. generated insights into potential reservoirs of diseases by using ML. Machine learning has also been used in the exploration of genome sequencing data. Dealing with large, complex, and hidden patterns can be daunting via other approaches, hence supporting the usefulness of machine learning as a plausible solution (17). Furthermore, sequencing data sequences has become increasingly easy to do compared to decades past, and largely attributed to the reduction in cost and the increase in throughput within veterinary and global health institutes. Put together, utilizing ML in whole genome sequencing improves source attribution (18), assessment of pathogenicity (19), prediction of antibiotic resistance phenotypes (20), and prediction of clinical outcomes (21). Simply put, an improved understanding of host-pathogen interaction and disease development and occurrence enhances molecular surveillance systems for most pathogens.

### 2.5 Artificial insemination

AI with its formidable data processing capabilities, assumes a pivotal role in the analysis of vast datasets encompassing animal reproductive physiology, genetics, and environmental factors. By assimilating and interpreting this wealth of information, AI algorithms become instrumental in identifying optimal breeding windows, thereby enhancing decision-making processes crucial for the success of artificial insemination procedures. Moreover, AI facilitates the development of predictive models that consider multifaceted parameters influencing reproductive success. This predictive modeling empowers farmers and veterinarians to anticipate fertility cycles, strategically select breeding techniques, and refine insemination protocols, ultimately contributing to the improvement of reproductive outcomes. In addition, AI-driven image recognition technologies play a crucial role in monitoring the reproductive health of animals. By analyzing visual cues such as estrus behavior, signs of heat, and overall reproductive status through images and videos, AI aids in fine-tuning the timing and approach of artificial insemination procedures. So far, AI has changed the game in the realm of animal artificial insemination, offering the capability to discern prolific dams and identify animals with the highest likelihood of success based on historical data. This application of AI represents a paradigm shift in precision breeding, utilizing advanced algorithms to analyze extensive datasets related to reproductive outcomes, genetic traits, and environmental factors. AI significantly enhances the efficiency of reproductive processes. The precision offered by AI minimizes errors in timing and technique, resulting in higher conception rates and improved overall reproductive efficiency in livestock breeding programs. Infertile and sub-fertile males can be effectively eliminated from breeding processes by utilizing semen analysis procedures such as Flow cytometry-based analysis and computer-assisted semen analysis (CASA) (22). CASA evaluates sperm count and motility in male animals, as well as structural morphology, in a clear and objective manner. Oftentimes, this system also gives a more accurate evaluation of the aforementioned qualities than light microscopy which has advanced over time with the ability to analyze sperm viability and DNA fragmentation, which are also markers of sperm quality. On the other hand, Flow cytometry-based semen analysis uses specific probes to examine those sperm characteristics. This biomarker-based semen analysis provides invaluable baseline data in the artificial insemination industry to improve the quality of semen from sires. Integration of AI into animal artificial insemination has and will continue to usher in a new era of precision breeding, redefining the landscape of animal agriculture. The strides made in reproductive efficiency, genetic optimization, and global accessibility to superior genetic resources underscore the profound impact of AI on shaping a more sustainable and advanced future for animal breeding and conservation efforts.

### 2.6 Patient assessment, treatment, and evaluation

Vital sign monitoring is an essential preliminary evaluation before disease diagnosis and treatment in animals. Real-time monitoring devices with AI-based analytics enable remote tracking of health indicators, allowing veterinarians to monitor conditions, identify early signs of deterioration, and optimize treatment. AI-driven decision support systems assist veterinarians in crafting customized treatment plans, considering factors such as the animal's genetics, medical history, response to medication, and susceptibility to anaphylaxis. This enhancement aids in selecting drugs, calculating doses, and assessing the effectiveness of different treatment approaches. Utilizing extensive data analysis and modeling, AI systems provide veterinarians with evidence-based recommendations and treatment plans adhering to global standards. AI enhances precision in decision-making by proposing personalized treatment options for each patient, drawing insights from scientific literature, analogous cases, and treatment results. In addition to being essential for diagnosis and prognostic assessment, long-term disease monitoring is also critical for assessing therapy outcomes. In the dairy sector, machine learning models have been studied, especially for the identification of diseases like mastitis (23) and lameness (24), prediction of calving time (25), and forecasting of milk production (26). AI assesses each animal's unique health profile, taking into account genetic predispositions, age, breed, and medical history. Utilizing all of this data, personalized treatment programs that are tailored to the unique requirements and traits of every patient are created. By evaluating the animal's reaction to therapy, this method optimizes pharmaceutical regimens and enables vets to modify dosages, take possible side effects into account, and fine-tune the treatment strategy based on current data and the unique characteristics of each patient. The battle against antimicrobial resistance (AMR) has been revolutionized in large part by AI. It finds people who are more likely to contract AMR, monitors the growth of resistant bacteria, looks for trends in the use of antibiotics, and quickly finds resistant infection outbreaks (27). The incorporation of AI in veterinary patient assessment, treatment, and evaluation has the potential to revolutionize veterinary medicine, offering more precise, efficient, and individualized care for animal patients. Continuous research and collaboration between veterinary professionals and AI experts will further refine and expand the applications of AI in veterinary practice.

### 2.7 Soft tissue and invasive surgery

Computer-enhanced visualization (CV) involves imaging and interpretation using ultrasonic machines. Units where CV is changing the game include image-based diagnosis and image-guided surgery (28). This CV-enhanced imaging has several applications spots in both human and veterinary medicine, including X-ray, ultrasound (US), computed tomography (CT), magnetic resonance imaging (MRI), positron emission tomography (PET) scans, retinal photography, dermoscopy, and histology, among others. A major bias that the application of AI and deep learning methods could remove is the subjectivity of image interpretation. Although several developments have been reported in the field of human medical image analysis over the past few decades (28), the use in veterinary clinical practice is still progressive, especially in low and middle-income regions of the world. Utilizing a blend of radionics and AI can be integrated into several medical imaging applications, including disease detection, characterization, and monitoring as seen in a study by Fraiwan and Abutarbush on predicting the need for surgery in horses presented with colic and the prognosis of those procedures (29). A Johns Hopkins University-designed “smart tissue autonomous robot” (STAR) has been demonstrated to perform human surgeons, especially in some surgical procedures such as bowel anastomosis in animals (3). AI-assisted surgeries would bolster their success rate essentially by planning surgical algorithms that identify optimal pathways, assess potential risks, and personalize surgical strategies based on individual patient characteristics, assisting surgeons in overlaying critical information on real-time imaging, enhancing precision and reducing the risk of errors and giving prompts or recommendations based on the ongoing procedure, helping surgeons make informed decisions and navigate unexpected challenges.

### 2.8 Limitations

Generally, there are several barriers to the widespread use of AI in diagnosis and treatment in Veterinary medicine. These include the absence of a credible reporting system that would require tens of millions of image or text samples that are not easily accessible, samples that are organized with inconsistent or scattered information (codes) that hinders the development of deep learning models, the fact that most models require labeled data for supervised learning, and the challenge of manually labeling data (30), at the individual level, work is not done and coordination with engineers and skilled labor is required (31). To attain the necessary efficacy, precision, and cost-effectiveness, the information technology business must make significant efforts. To further the adoption and use of electronic health records, laboratories, health centers, and veterinary institutions must collaborate (31). In animal healthcare, there are no privacy laws guiding the use of patient data, but human healthcare is governed by stringent data privacy regulations, such as the General Data Protection Regulations (GDPR) in the European Union and the Health Insurance Portability and Accountability Act (HIPAA) in the United States. These regulations mandate secure storage, transmission, and access control for patient health data, ensuring privacy and confidentiality. AI algorithms in human healthcare benefit from systematically collected and well-annotated data, adhering to the FAIR Guiding Principles. This systematic data collection follows standards like Systematized Nomenclature of Medicine—Clinical Terminology (SNOMED-CT) which has been adopted by data repositories such as Veterinary Medical Database (VMDB), Small Animal Veterinary Surveillance Network (SAVSNET). However, retrieving and analyzing the data can be challenging because they are mostly free texts that are not easily “queryable” (32). AI has limits when it comes to veterinary practice and research. Both the volume and quality of the data that is accessible greatly influence AI systems. Because veterinary medicine treats a wide variety of animal species with different anatomies, physiologies, and genetic origins, it can be difficult to gather big and diverse datasets for use in clinical practice. Species categorization would be required. It can be difficult to create universal AI models that consider this heterogeneity, and various species may require distinct models. Large language models (LLM) have the potential to serve as an effective method for veterinary information extraction since they can be adapted to different tasks and have a large number of parameters and get structured information from unstructured reports. Careful thought must be given to issues of permission which is usually from the pet owners, data privacy of both pets and owners, and responsible AI use in veterinary practice. A lack of consistency due to unstructured veterinary data may result in misdiagnoses and mistreated patients, as well as make it challenging to monitor patients' improvement over time. However, Text mining and information extraction are two examples of Natural Language Processing (NLP) approaches that can process unstructured veterinary records to extract pertinent clinical data, boosting accessibility and easing the training of AI models. These methods can be especially helpful in addressing the difficulties posed by unstructured veterinary data, such as the absence of defined forms for veterinary reports. Combined language collected from veterinary reports, can be subjected to text mining methods which facilitate the analysis and extraction of pertinent data, and then through information extraction, veterinary reports which contain specific clinical terminology and concepts, such as animal species, diagnoses, and therapies, can be identified and extracted using information extraction algorithms. NLP techniques such as sentiment analysis and epidemiological surveillance can be employed to examine the sentiment or emotion conveyed in veterinary reports, offering valuable insights into staff attitudes and patient outcomes. In addition to conventional epidemiological monitoring techniques, text mining can be utilized to retrieve pertinent information from veterinary forums and other internet sources. Moreover, antimicrobial consumption in clinical records may be highly accurately matched by NLP algorithms, which makes it easier to track and comprehend antimicrobial usage in veterinary practice. The veterinary field may increase accessibility, simplify the training of AI models for a range of uses, including diagnosis, treatment planning, and epidemiological surveillance, and extract useful clinical data from unstructured reports by utilizing NLP approaches.

LLMs are one type of language model that can be used to generate structured reports or summaries based on important discoveries from unstructured veterinary data. Large veterinary report datasets are used to train LLMs, allowing them to automatically produce structured reports or summaries based on important discoveries from unstructured data. This can help AI algorithms even more by enhancing data consistency and organization. LLMs have the capability to extract pertinent data from veterinary records, including animal species, diagnoses, and treatments. These data can then be compiled into structured summaries or reports that provide easy analysis and comparison between various veterinary practices. This can simplify large-scale investigations and epidemiological surveillance in addition to helping to increase the accuracy of diagnosis and treatment. LLMs can also be used to find trends and patterns in veterinary data, which can enhance patient outcomes and guide clinical judgment. Put together, the structure and accessibility of veterinary data can be greatly enhanced using LLMs in report production, which will make it easier to construct AI models for a range of veterinary medicine applications. Furthermore, maintaining ethical standards is made more difficult by the absence of defined regulatory frameworks for AI uses in veterinary practice. Additionally, there may be differences in the acceptance of AI-driven diagnosis and therapy by clients. To allay pet owners' worries about trust and the human-animal link, veterinarians must adequately explain to them the role AI plays in veterinary care. Collaboration between data scientists, AI specialists, and veterinarians is necessary for the successful application of AI in veterinary practice. The veterinary medicine-specific datasets needed for the AI models might be scarcer than the human healthcare datasets. The creation of precise and trustworthy AI models for veterinary applications as well as the smooth integration of AI technologies into veterinary workflows may be hampered by the lack of data and interdisciplinary collaboration. Even though AI has a lot of potential to change veterinary practice and research, appropriate and successful inclusion into the field depends on addressing these constraints. Overcoming these obstacles and optimizing the advantages of AI in veterinary medicine require continued research and cooperation.

## 3 The role of artificial intelligence in veterinary/biomedical research

### 3.1 Antimicrobial resistance research

The emergence of resistance in microbes poses a significant threat to world health, particularly in underdeveloped nations with inadequate healthcare infrastructure. As such, it is critical to create instruments to facilitate the quick development of novel medications, vaccines, and diagnostic instruments. AI has the potential to have a substantial influence on antimicrobial resistance and the detection of antibiotic residue in food, such as meat and milk, as existing laboratory approaches are time-consuming and fraught with difficulties. The current use of laboratory techniques is time demanding with lots of challenges, therefore, AI has the potential to significantly impact antimicrobial resistance and also detection of antibiotic residue in food such as meat and milk. AI can analyze large datasets to identify potential drug targets and design new antibiotics with enhanced efficacy and reduced resistance potential. Some of the AI-powered algorithms can analyze surveillance data to track antimicrobial resistance trends, identify emerging threats, and inform public health interventions (33). Specific AI tools have been developed for AMR research such as Naïve Bayes for predicting antibiotic resistance and identifying the key factors of resistance, decision trees for estimating the burden of AMR and guiding antibiotic use, random forest for predicting antibiotics combination, support vector machine for designing new antibiotics (34). AI has been used in developing novel strategies for Antimicrobial Susceptibility testing and Whole Genome Sequencing. These new methods are faster and more accurate than the traditional way and have the potential to change and enhance the way AMR is diagnosed and treated (34). A new antibiotic should ideally be developed throughout 10–15 years, but the advent of AI may allow for a development time of only 2 years on average (35). This would represent a significant advancement in the field of antimicrobial resistance research. The use of AI in identifying patients at high risk of antimicrobial resistance (AMR) infection, tracking the transmission of resistant bacteria, monitoring antibiotic consumption patterns, and detecting resistance infection outbreaks has revolutionized the fight against AMR (27). Consumption of antibiotic residue in food animals, such as meat, eggs, and milk, causes humans to develop antibiotic resistance. Therefore, the application of AI in livestock production to detect and control the use of antibiotics in livestock will have a great impact in curbing the menace of antibiotic resistance and also will guide researchers and scientists in tracking the spread of resistance genes. In food substances especially meat and dairy industry, it can be applied to detect antibiotic residue in food and also track the source of such food. Therefore, AI is a promising tool or technology with the potential to make a significant impact in AMR research in livestock and humans, further development of AI-powered in the future will provide an innovative technology to combat AMR.

### 3.2 Cancer research

Cancer is still a dangerous illness that affects both people and pets. Despite its severity, medication, radiation, and surgical techniques have all been investigated by researchers as potential treatments. Finding novel approaches to treating cancer cells and enhancing diagnostic techniques is therefore imperative. Cancer research is being revolutionized by AI in veterinary medicine. AI-powered instruments are essential for improving cancer detection, management, and prevention. There are a number of AI predictive models that can determine risk factors and estimate a person's chance of getting cancer. Table 1 presents a list of useful webservers and tools for diseases in animals and humans. These models are essential for facilitating early detection and putting intervention plans into action. A study was conducted where an AI-powered tool autonomously identified and delineated suspicious areas of prostate cancer without requiring any human intervention. Therefore, the AI tool proves to be a promising asset in cancer imaging, significantly aiding radiologists in achieving accurate cancer diagnoses (10). Moreover, its pivotal role extends to the identification of cancer drug targets. The tool contributes to predicting the efficacy and toxicity of these drug candidates, while also optimizing treatment regimens. This optimization is achieved through the thorough analysis of extensive cancer genomic and proteomic datasets, showcasing the broad and impactful applications of AI in advancing cancer research and treatment (39). This represents a substantial advancement, as it has the potential to expedite the drug discovery process and uncover novel cancer drug targets that traditional methods might overlook. Furthermore, it has paved the way for personalized treatment plans tailored to individual patients. By analyzing the specific characteristics of their tumors, the AI tool can predict how patients might respond to various therapies, marking a significant stride toward more effective and personalized cancer treatment strategies (40). These advancements indeed offer significant promise in enhancing patient outcomes and alleviating the burden of cancer. Notably, several models have been devised to facilitate early cancer diagnosis. In a particular study, researchers employed an algorithm to identify cancerous abnormal growths on CT scans. The algorithm, rooted in AI and utilizing Radiomics, demonstrated the capacity to extract crucial information from medical images that might prove challenging for the human eye to discern. This demonstrates how AI may be used to increase the accuracy and efficacy of cancer diagnosis, especially in the early stages when prompt intervention can significantly alter the course of treatment (41). Although low-dose computed tomography (LDCT) has become a useful technique for screening for lung cancer, false-positive rates remain a problem. AI-driven technologies are being used to improve the precision of lung cancer tests and lower the number of false positives in order to solve this problem. These AI tools use sophisticated algorithms and image processing to increase the accuracy of identifying possible lung cancer symptoms, leading to more accurate and consistent screening outcomes. In a recent study published, a deep learning model called Sybil was developed and validated to predict future lung cancer risk from a single LDCT scan (42). This model plays a crucial role in pinpointing individuals with a heightened risk of lung cancer. It enables more focused screening and preventive measures, minimizing unnecessary screenings for those at lower risk. This approach optimizes resource allocation in healthcare and reduces potential harm from excessive radiation exposure, emphasizing the importance of personalized and efficient lung cancer screening practices.

**Table 1:** Some examples of useful tools and available webservers dedicated to the prediction of zoonotic/veterinary diseases and monitoring.

**Tool name**	**Year of publication**	**Description/application**	**GitHub/website link**
ZOVER	2014 (36)	Database of zoonotic and vector-borne viruses which incorporates virological, ecological, and epidemiological data for better understanding of those pathogens.	http://www.mgc.ac.cn/cgi-bin/ZOVER/main.cgi
IHBDP	2019, 2022 (37)	The Integrated Health Big Data Platform compiles medical data from hospitals, e-health records, and vaccination records. Reportedly used in identifying Dengue and Tuberculosis (TB) patients.	NA
FluSPred	2022 (13)	Flu Spread Prediction is a machine learning-based tool which can predict human related Influenza viral strains by targeting their protein and genome sequences, accurately predicting the zoonotic potential of the viral strain.	https://webs.iiitd.edu.in/raghava/fluspred/index.html
P-HIPSTer	2019	(Pathogen-Host Interactome Prediction using STructurE similaRity) is an algorithm which utilizes sequence- and structure-based information to extrapolate interactions between pathogens and human proteins.	http://phipster.org/
WIsH	2019 (38)	WIsH helps in predicting the prokaryotic hosts of phages by assessing their genomic sequences.	https://github.com/soedinglab/WIsH
VIDHOP	2021 (12)	VIDHOP is a virus-host predicting tool. It has been specifically used for Influenza A virus, rabies lyssavirus and rotavirus A predictions.	https://github.com/flomock/vidhop
VirHostMatcher	2020	A network-based computational tool for predicting virus-host interactions. Specifically used in viral-host matching based on oligonucleotide frequency (ONF) comparison.	https://github.com/WeiliWw/VirHostMatcher-Net
BlueDot	2013	AI-powered platform employed in tracking and predicting the spread of infectious diseases. Reportedly predicted Zika virus spread to Florida in 2016 and the movement of the 2014 Ebola outbreak out of West Africa.	https://bluedot.global/
EPIWATCH	2020	AI-driven system harnessing vast, open-source data to generate automated early warnings for epidemics worldwide. Contains full language and geographic information system capability. Efficient in early identification outbreak signals.	https://www.epiwatch.org/

### 3.3 Genomic and vaccine development research

AI is rapidly becoming a transformative methodology in the field of genomic research, offering a powerful toolkit for analyzing vast complex genomic datasets and extracting meaningful insights. Artificial algorithms, particularly machine learning techniques, excel at identifying patterns and relationships in data that may elude traditional methods, enabling researchers to make novel discoveries and accelerate the pace of genomic research. One of the key roles of AI in genomic research is variant calling and annotation (43). The AI algorithm can effectively identify and characterize Single Nucleotide and Polymorphisms (SNPs), Insertions and Deletions (Indels), and other functional and structural variants, this is particularly crucial for Whole Genome Sequencing (WGS) data, which generates data that require sophisticated analysis tools to identify and interpret genetic variants. AI also plays a critical role in variant impact prediction, assessing the potential functional consequences of genetic variants (43). By analyzing pattern variations across populations and linking variants to phenotypic traits, the AI algorithm can predict the likelihood that a particular variant may contribute to disease or other traits of interest. This information is invaluable for prioritizing variants for further functional studies and developing personalized medicine approaches. AI is being employed to improve phenotype-genotype mapping, the process of associating genetic variants with specific traits or diseases (43). This algorithm can identify complex relationships between genetic variants and phenotypes, even when these relationships are subtle or involve multiple genes. This capability is essential for exploring the genetic underpinnings of complex diseases and developing effective diagnostic and therapeutic strategies. AI plays an important role in vaccine design and development research. The rapid development of vaccines against the SARS-CoV-2 virus, responsible for the COVID-19 pandemic has highlighted the transformative potential of AI in vaccine research. AI is a powerful tool for accelerating the identification, design, and evaluation of vaccine candidates, promising to revolutionize the fight against infectious diseases. This AI can analyze large biological datasets, including genomic and proteomic information, to pinpoint potential antigens with high immunogenic potential (44). AI can also predict the binding affinities between antigens and immune molecules, guiding the selection of antigens that effectively trigger a protective immune response (45). The AI-powered tool also can optimize vaccine design including the selection of adjuvants, and substances that enhance the immune response to the vaccine and analyze complex datasets to identify adjuvants that synergistically interact with antigens, potentiating vaccine efficacy. This data-driven approach allows for the development of more potent and durable vaccines. AI can predict the efficacy and safety of potential vaccine candidates before they enter clinical trials. On the other hand, machine learning algorithms can analyze historical data and identify patterns that correlate with vaccine efficacy and safety outcomes. These predictions guide the selection of candidates for further development, reducing the time and resources invested in testing ineffective or unsafe vaccines.

### 3.4 Phytomedicinal, ethnomedicinal, and pharmaceutical research

AI could improve drug discovery and the development of processes from natural products. Novel drug development is complex, time-consuming, and labor-intensive. The use of methods and techniques such as trial and error experimentation and high throughput screening are a few of the reasons for the limitations of drug development (46). However, AI plays a vital role in accelerating the timeline via effective and efficient analysis of vast datasets (47). AI-powered tools can generate and evaluate millions of potential drug candidates, significantly reducing the time and effort required for drug design, it also can identify novel drug targets with potential therapeutic efficacy (43). AI can potentially change the drug discovery process by accelerating the identification of promising drug candidates, optimizing clinical trial design, and enabling personalized medicine approaches. AI-powered models can predict the efficacy and potential adverse effects of drug candidates, allowing researchers to prioritize the most promising compounds for further development and reduce the number of failed clinical trials (46). Additionally, AI can facilitate the development of personalized medicine approaches by tailoring drug treatments to individual patient's characteristics maximizing therapeutic efficacy, and minimizing adverse effects. AI can optimize clinical trial designs by selecting the most suitable population of patients, treatment regimens, and other outcome measures leading to more efficient and effective drug development (46).

## 4 Conclusion and future perspective

AI stands at the forefront of addressing pressing global health challenges across various domains. In the realm of AMR, AI offers a transformative solution by analyzing extensive datasets to identify drug targets, design new antibiotics, and guide antibiotic use, thereby expediting the development of vital tools for combating AMR. In the context of cancer research, AI's role in enhancing diagnostic methods, treatment strategies, and drug discovery holds promise for improving patient outcomes. The interconnected nature of zoonotic and reemerging diseases requires innovative approaches, and AI in health and veterinary research emerges as a pivotal tool for early detection, containment, and accurate diagnostics, as evidenced by its impact during the recent COVID-19 pandemic. Genomic research benefits significantly from AI's capabilities in variant calling and annotation, contributing to personalized medicine approaches. Lastly, AI's influence in drug discovery and development is marked by its ability to streamline processes, identify novel drug targets, and optimize clinical trial designs, paving the way for more efficient and effective therapeutic solutions. The integration of AI across these diverse healthcare domains reflects its transformative potential in revolutionizing the landscape of medical research and application. There are a number of challenges inherent in veterinary diagnostic imaging data sets. First, dealing with the variability of the species and breeds seen in veterinary practice and the usual high patient caseload resulting in the collection of large data can be a daunting task. Second, the dearth of common examples of emerging diseases for algorithm training is a major setback, therefore certain diagnoses may not be captured if such examples are not considered beforehand. The use of veterinary databases such as VMDB should be encouraged amongst veterinary personnel and professionals as it would be a basis for training AI algorithms for veterinary practice and research based on available data structures and images. Even though there is a growing interest and efforts in incorporating AI for utilization in human medical radiology, there is still a big knowledge gap for a successful full adoption of the same in veterinary medicine (48). A plausible method would be a combination of new and conventional methodologies into a hybrid solution that could help in a smoother transition to newer, powerful, and useful predictive systems easily adoptable by practitioners and researchers alike (49–51).

## Data availability statement

The original contributions presented in the study are included in the article/supplementary material, further inquiries can be directed to the corresponding author.

## Author contributions

OA: Conceptualization, Writing—original draft, Writing—review & editing. II: Writing—original draft, Writing—review & editing. VA: Writing—original draft, Writing—review & editing. SS: Writing—review & editing. OB: Writing—review & editing. SO: Writing—review & editing. MO: Writing—review & editing. DO: Writing—review & editing. CU: Writing—review & editing. IO: Writing—review & editing. JG: Writing—review & editing. QA: Writing—review & editing. PB: Writing—review & editing. SR: Writing—review & editing. KS: Writing—review & editing.
